# O Estudo ELSA-Brasil e a Atividade Física na Proteção Contra o Desenvolvimento de Diabetes Tipo 2

**DOI:** 10.36660/abc.20250464

**Published:** 2025-09-04

**Authors:** Rodrigo Delevatti, Larissa dos Santos Leonel, Leandro Franzoni

**Affiliations:** 1 Universidade Federal de Santa Catarina Florianópolis SC Brasil Universidade Federal de Santa Catarina, Florianópolis, SC – Brasil; 2 Universidade Federal do Rio Grande do Sul Porto Alegre RS Brasil Universidade Federal do Rio Grande do Sul, Porto Alegre, RS – Brasil

**Keywords:** Diabetes Mellitus, Exercício Físico, Epidemiologia

O diabetes mellitus tipo 2 (DMT2) é uma doença de alta prevalência,^1^ considerada um grande problema de saúde pública, que implica em custos pessoais, bem como representa um expressivo fardo financeiro aos serviços de saúde.^1 , 2^ Em sua abordagem preventiva e terapêutica, o papel protetor da educação física tem sido estabelecido, porém a otimização dos seus benefícios, sobretudo analisando a relação de características inerentes à prática de atividade física com o desenvolvimento do DMT2 é algo ainda em aberto.

Nesse cenário, o artigo "Associação Dose-Resposta entre Trajetórias de Intensidade da Atividade Física no Lazer e Diabetes entre Homens e Mulheres no ELSA-Brasil"^3^ colabora significativamente, acompanhando uma amostra expressiva de indivíduos, de diferentes locais no Brasil, testando a associação entre diferentes doses de atividade física no tempo de lazer e as condições de pré-diabetes e DMT2 em adultos e idosos. Como exposição, os diferentes perfis de prática de atividade, considerados aqui como doses (produto da frequência, duração e intensidade), sendo classificados em fraco, moderado e forte, termos que fazem alusão à intensidade de esforço.

Caracterizando melhor o estudo em discussão, trata-se de um desenho longitudinal, com caráter epidemiológico associativo, conduzido em cinco universidades públicas e um instituto de pesquisa, contabilizando mais de 10.000 participantes, com aproximadamente 20% da amostra classificada com DMT2, com maioria do sexo masculino. De modo geral, os achados apontam maior prática de atividade física no sexo masculino, em todos os estratos de intensidade avaliados, e a alta intensidade como protetora do desenvolvimento de DMT2. Como achados gerais, foram os homens aqueles que mais praticavam atividade física, em todas as intensidades.

Mais especificamente, as análises associativas indicaram a prática de atividade física em intensidade forte reduzindo [OR=0,63 (IC95% 0,40-0,98) homens e OR=0,33 (IC95% 0,14-0,79) mulheres] a chance de desenvolver DM2, tendo como referência de comparação a intensidade moderada. Ainda, no público masculino, a prática em intensidade fraca implica em uma chance maior (OR=1,36 [IC95% 1,09-1,69]) do desenvolvimento de pré-diabetes, também em relação aos praticantes de atividade física moderada.

A importante evidência de proteção metabólica pelas maiores intensidades deve ser tratada com cautela, tendo em vista a natureza das análises, pois o viés de causalidade reversa pode se fazer presente, uma vez que, especialmente em atividades com suporte do próprio peso, como caminhada e corrida, indivíduos com pré-diabetes ou DMT2 podem ter dificuldades de realizar as maiores intensidades. Nessa direção, os achados do referido estudo reforçam a importância da intensidade na prática de atividade física como estratégia preventiva no desenvolvimento do DMT2. Contudo, todos aqueles que promovem ou desejam promover atividade física com esse fim devem considerar que a maior incidência das condições em análise ocorre entre meia idade e terceira idade, em pessoas que costumam apresentar condição de sobrepeso ou obesidade associada a disfunções osteomioarticulares, sendo barreiras para práticas físicas, sobretudo intensas, antes mesmo do diagnóstico.^4^ Ainda, considerando o fato de que as mulheres quando praticam atividade física em intensidade mais forte, mesmo que em menor proporção que os homens, apresentam maiores benefícios, se destaca a necessidade de estratégias mais direcionadas e equitativas para a promoção de atividade física no lazer, considerando gênero, condição clínica e funcional.

Com base em um delineamento longitudinal robusto, com utilização de ferramenta de fácil aplicabilidade e baixo custo para verificar a prática de atividade física, em uma amostra ampla, há a limitação natural de estudos epidemiológicos da não caracterização precisa de domínios fisiológicos e modalidades praticadas. Por considerar diferentes constructos como frequência, duração e intensidade, os resultados obtidos não se restringem apenas a intensidade fraca, moderada ou forte, mas compreendem doses de diferentes magnitudes, como o próprio título indica, sendo estudo de dose-resposta. Esta compreensão composta de dose, em que a intensidade faz parte do trabalho realizado, aproxima os achados de estudos prévios que apontaram variáveis de volume, como duração semanal,^5^ frequência semanal e número de séries^6^ e progressão de carga^7^ como variáveis importantes para o controle glicêmico.

Reforçando a discussão, os autores citam o estudo de Alvarez et al.^8^ pela sua eficácia usando o HIIT em mulheres com DMT2. Este, como também recentemente evidenciado por Garcia et al.^9^ reforçam o HIIT e consequentemente a intensidade alta como importantes no controle glicêmico, porém no estudo de Alvarez et al.,^8^ como muitos de HIIT nesse público, existe substancial progressão de variáveis de volume ao longo da intervenção, como aumento do número de estímulos, do tempo de estímulos e da densidade (relação estímulo: recuperação), aumentando consideravelmente o tempo total de sessão ao longo de 16 semanas. Esse olhar ampliado sobre as intervenções indica que se considere a importância da intensidade para o metabolismo glicêmico, mas em um olhar associado com a quantidade e a manipulação da prática ao longo do tempo.

Diante disso, estudos futuros devem buscar delineamentos que isolem e equalizem variáveis de carga, especialmente o volume e a intensidade, para verificar de forma mais precisa se a intensidade, de fato, é um preditor independente de proteção contra o desenvolvimento do DMT2. Investigações que estratifiquem os efeitos por tipo de exercício (aeróbio, resistido, combinado) podem elucidar quais contextos e modalidades oferecem maior benefício protetivo, avançando ainda mais nessa importante temática, na qual este estudo do Elsa-Brasil, nos convida brilhantemente a avançar.
