# Supplementary material for: O Estudo ELSA-Brasil e a Atividade Física na Proteção Contra o Desenvolvimento de Diabetes Tipo 2
Source: Arq Bras Cardiol. 2025 Sep 4;122(8):e20250464. [Article in Portuguese] doi: 10.36660/abc.20250464 (PMC12671593; doi:10.36660/abc.20250464)
Supplement: Translation

# Translation: The ELSA-BRASIL Study and Physical Activity in Protecting Against the Development of Type 2 Diabetes

**DOI:** 10.36660/abc.20250464i

**Authors:** Rodrigo Delevatti, Larissa dos Santos Leonel, Leandro Franzoni

**Affiliations:** 1 Florianópolis SC Brazil Universidade Federal de Santa Catarina, Florianópolis, SC – Brazil; 2 Porto Alegre RS Brazil Universidade Federal do Rio Grande do Sul, Porto Alegre, RS – Brazil

**Keywords:** Diabetes Mellitus, Exercise, Epidemiology

Type 2 diabetes mellitus (T2DM) is a highly prevalent disease,^1^considered a major public health problem, which implies personal costs, as well as representing a significant financial burden on health services.^1 , 2^In its preventive and therapeutic approach, the protective role of physical education has been established, but the optimization of its benefits, especially analyzing the relationship between characteristics inherent to the practice of physical activity and the development of DMT2, is still open.

In this scenario, the article "Dose-Response Association between Leisure-Time Physical Activity Intensity Trajectories and Diabetes among Men and Women in ELSA-Brasil"^3^collaborates significantly, following a significant sample of individuals from different locations in Brazil, testing the association between different doses of leisure-time physical activity and pre-diabetes and T2DM in adults and older adults. The exposure is based on the different activity profiles, considered here as doses (the product of frequency, duration, and intensity), classified as low, moderate, and high, terms that refer to the intensity of the effort.

To better characterize the study under discussion, it is a longitudinal design with an associative epidemiological approach, conducted at five public universities and a research institute, totaling over 10,000 participants, with approximately 20% of the sample classified as having T2DM, the majority of whom were male. Overall, the findings indicate greater physical activity among men, across all intensity levels evaluated, and high-intensity exercise is protective against the development of T2DM. Overall, men were the ones who engaged in the most physical activity, at all intensities.

More specifically, associative analyses indicated that practicing high-intensity physical activity reduced [OR=0.63 (95%CI 0.40-0.98) men and OR=0.33 (95%CI 0.14-0.79) women]the chance of developing DM2, using moderate intensity as a reference for comparison. Furthermore, in the male population, practicing at low intensity implies a greater chance (OR=1.36 [95%CI 1.09-1.69]) of the development of pre-diabetes, also in relation to those who practice moderate physical activity.

The significant evidence of metabolic protection from higher intensities should be treated with caution, given the nature of the analyses. Reverse causality bias may be present, as individuals with pre-diabetes or T2DM may have difficulty performing higher intensities, especially in weight-bearing activities such as walking and running. In this sense, the findings of this study reinforce the importance of intensity in physical activity as a preventive strategy for the development of T2DM. However, all those who promote or wish to promote physical activity for this purpose should consider that the highest incidence of the conditions under analysis occurs between middle and old age, in people who are often overweight or obese, associated with musculoskeletal disorders, which pose barriers to physical activity, especially intense ones, even before diagnosis.^4^Furthermore, considering the fact that women, even if to a lesser extent than men, experience greater benefits when they engage in higher-intensity physical activity, highlights the need for more targeted and equitable strategies for promoting leisure-time physical activity, considering gender, clinical status, and functional status.

Based on a robust longitudinal design, using an easy-to-apply, low-cost tool to assess physical activity in a large sample, epidemiological studies are naturally limited by the lack of precise characterization of physiological domains and types of activity. Because they consider different constructs such as frequency, duration, and intensity, the results obtained are not limited to low, moderate, or high intensity, but encompass doses of varying magnitudes, as the title suggests, making this a dose-response study. This composite understanding of dose, in which intensity is part of the work performed, aligns with the findings of previous studies that identified volume variables such as weekly duration,^5^weekly frequency and number of sets,^6^and load progression^7^as important variables for glycemic control.

Reinforcing the discussion, the authors cite the study by Alvarez et al.^8^for its effectiveness using HIIT in women with T2DM. This, as also recently demonstrated by Garcia et al.,^9^reinforces HIIT and consequently high intensity as important for glycemic control. However, in Alvarez's study,^8^like many HIIT studies in this population, there is a substantial progression of volume variables throughout the intervention, such as an increase in the number of stimuli, stimulus duration, and density (stimulus:recovery ratio), considerably increasing the total session time over 16 weeks. This expanded view of interventions indicates that the importance of intensity for glycemic metabolism should be considered, but in conjunction with the quantity and manipulation of the practice over time.

Therefore, future studies should seek designs that isolate and equalize load variables, especially volume and intensity, to more accurately determine whether intensity is, in fact, an independent predictor of protection against the development of T2DM. Research that stratifies the effects by type of exercise (aerobic, resistance, combined) can elucidate which contexts and modalities offer the greatest protective benefit, further advancing this important topic, which this Elsa-Brasil study brilliantly invites us to pursue.
